# Gross Total vs. Subtotal Resection on Survival Outcomes in Elderly Patients With High-Grade Glioma: A Systematic Review and Meta-Analysis

**DOI:** 10.3389/fonc.2020.00151

**Published:** 2020-03-18

**Authors:** Qian Han, Hengpo Liang, Peng Cheng, Hongjie Yang, Pingfan Zhao

**Affiliations:** ^1^Department of Radiotherapy, Henan Provincial People's Hospital, Zhengzhou, China; ^2^Department of Outpatient, Henan Provincial People's Hospital, Zhengzhou, China

**Keywords:** high-grade glioma, elderly patients, gross total resection, subtotal resection, mortality, meta-analysis

## Abstract

**Background:** The optimal strategy for the management of high-grade glioma in the elderly (≥60.0 years) remains controversial, especially regarding the effects of surgical extent on survival outcomes. The purpose of this study was to compare gross total resection (GTR) with subtotal resection (STR) for treatment effects in elderly patients with high-grade glioma.

**Methods:** Three electronic databases were systematically searched, including PubMed, EmBase, and the Cochrane library, from inception to August 2018. Hazard ratios (HRs) or odds ratios (ORs) with corresponding 95% confidence intervals (CIs) were used to express summary effect estimates using the random-effects model. Nineteen retrospective observational studies involving a total of 10,815 elderly patients with high-grade glioma were included in this meta-analysis.

**Results:** The summary results indicated that GTR was associated with a significant improvement in overall survival (OS) compared with STR (HR = 0.70, 95% CI = 0.64–0.77). In addition, elderly patients administered GTR showed lower risk of 3-month mortality (OR = 0.47, 95% CI = 0.24–0.93), 6-month mortality (OR = 0.38, 95% CI = 0.26–0.56), 9-month mortality (OR = 0.35, 95% CI = 0.25–0.49), and 1-year mortality (OR = 0.40, 95% CI = 0.29–0.56). Pooled OS data differed when stratified by publication year, country, sample size, disease status, and study quality.

**Conclusion:** GTR seems to be more effective than STR in achieving longer survival in elderly patients with high-grade glioma.

## Introduction

Glioblastoma multiforme (GBM) is the most frequent malignancy of the central nervous system, with an incidence of approximately 4.8/100,000 cases annually (1, 2). Despite standard treatments used for GBM, including surgery, radiotherapy, and temozolomide, the survival outcomes remain poor, with a median survival of 14–17 months (2, 3) and a 5-year survival rate of just 10% (4, 5). Currently, the treatment options for recurrent cases include systemic, re-irradiation, and second surgery in order to improve outcomes. The risks and benefits of radiation, chemotherapy, and surgical extent in GBM patients have been assessed in numerous studies (6–9). However, the value of surgical extent in elderly patients with high-grade glioma remains unestablished.

Gross total resection (GTR) is defined as the removal of all tumors, as gauged by magnetic resonance imaging. Mounting evidence indicates that aggressive cytoreductive surgery is associated with significantly improved survival outcomes, which could be due to surgery being influenced by the mutational status of the isocitrate dehydrogenase (IDH) gene (10–12). A previous meta-analysis based on 37 studies assessed whether greater extent of surgery affects survival outcomes in GBM patients and found that GTR is associated with significantly improved overall survival (OS) and progression-free survival (PFS) compared with subtotal resection (STR) (13). However, GTR and STR have not been comparatively assessed for their effects in elderly patients with high-grade glioma.

Numerous studies have been performed in elderly patients with high-grade glioma aiming to evaluate the effect of surgical extent on survival outcomes and reported inconsistent results. Clarifying whether GTR could offer greater survival benefits compared with STR is particularly important for elderly patients with high-grade glioma as it remains undetermined. Therefore, this meta-analysis of published studies aimed to comparatively evaluate the therapeutic effects of GTR and STR in elderly patients with high-grade glioma.

## Materials and Methods

### Data Sources, Search Strategy, and Selection Criteria

This review was conducted and reported according to the Preferred Reporting Items for Systematic Reviews and Meta-Analysis Statement issued in 2009 (14). A comprehensive electronic literature search was performed in the PubMed, EmBase, and the Cochrane library databases from inception to August 2018, with the following text word or Medical Subject Heading terms: (“high grade glioma” OR “malignant astrocytoma” OR “malignant oligodendroglioma” OR “glioblastoma multiforme” OR “oranaplastic astrocytoma” OR “oranaplastic oligodendroglioma”) AND (“gross total” OR “subtotal” OR “partial” OR “extent of resection”). The reference lists of all retrieved studies and relevant review articles were manually searched to identify any new eligible studies.

The literature search and study selection were independently carried out by two reviewers, and any disagreement was resolved by group discussion until a consensus was reached. Studies were included if they met the following criteria: (1) patients were elderly individuals (≥60.0 years old) with high-grade glioma; (2) the intervention and control groups were administered GTR and STR, respectively; (3) at least one of the following outcomes were reported, including OS and 3-month, 6-, 9-, and 1- mortality; and (4) study design as prospective, retrospective, or case series.

### Data Collection and Quality Assessment

The data items extracted included the first author's surname, publication year, country, study design, sample size, age range, male percentage, and investigated outcomes. Study quality was assessed by the Newcastle–Ottawa Scale (NOS), which is a comprehensive tool for evaluating the methodological quality of observational studies (15). Moreover, the NOS is based on selection (four items), comparability (one item), and outcome (three items), with a “star system” ranging from 0 to 9. Data extraction and quality assessment were performed by two reviewers, and inconsistent results were adjudicated by the corresponding author referring to the original studies.

### Statistical Analysis

The STATA 10.0 software (Stata Corporation, College Station, TX, USA) was employed to assess OS using hazard ratios (HRs); mortality rates at different follow-up periods were expressed as odds ratios (ORs) with corresponding 95% confidence intervals (CIs). The summary results for OS and 3-, 6-, 9-month, and 1-year mortality were assessed by the random-effects model (16, 17). Heterogeneity among the included studies for the investigated outcomes was assessed by the *I*^2^ test and *Q* statistic; *I*^2^ > 50.0% or *P*-value for *Q* statistic < 0.010 were considered to indicate significant heterogeneity (18, 19). Sensitivity analyses were performed for the investigated outcomes by sequential exclusion of individual studies (20). Subgroup analyses were carried out for OS and 3-, 6-, 9-month, and 1-year mortality according to publication year, country, sample size, age criteria, male percentage, and study quality. *P*-values between subgroups were also assessed by the chi-square test and meta-regression (21). Publication bias was qualitatively assessed by funnel plots and quantitatively by Egger (22) and Begg tests, respectively (23). *P*-values for pooled results were two-sided. *P* < 0.005 was considered statistically significant.

## Results

### Literature Search

**Figure 1:**
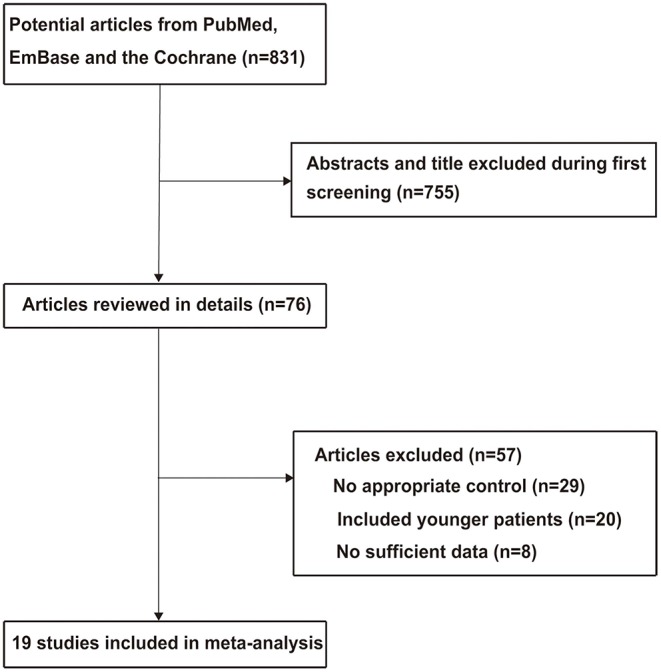
Flowchart of the literature search and study selection processes.

The literature search and study selection processes are detailed in Figure 1. The initial search from the three electronic databases yielded 831 citations. Seven hundred and thirty-one reports were excluded as duplicates or for studying irrelevant topics by reading the titles and abstracts. A total of 76 studies were retrieved for full-text evaluation, and 57 were further excluded due to the following reasons: no appropriate control (*n* = 29), inclusion of younger patients (*n* = 20), and no sufficient data (*n* = 8). Therefore, 19 studies met our inclusion criteria and were selected for final meta-analysis (24–42). There were no additional eligible studies from the manual search of the reference lists of these studies.

### Characteristics

The general characteristics of the included studies are shown in Table 1. All the included studies had a retrospective observational design and were published from 1998 to 2018. The sample sizes ranged from 10 to 8,152 patients in individual studies, and male percentages were 37.5–70.0%. Eight studies were conducted in America, eight were performed in Europe, and the remaining three studies were carried out in Asia. Five studies used ≥70.0 years of age as the cutoff, and the remaining 14 used 60–69 years of age as the cutoff. One study specifically included patients with anaplastic gliomas. Eight, six, and five studies had scores of 7, 6, and 5, respectively.

**Table 1:** Baseline characteristics of the included studies.

**References**	**Country**	**Study design**	**Sample size**	**Age range (years)**	**Percentage male**	**Study quality**
Mohan et al. (24)	Columbia	Retrospective	49	≥70.0	NA	5
Combs et al. (25)	Germany	Retrospective	29	≥65.0	67.4	6
Stummer et al. (26)	Germany	Retrospective	120	≥60.0	63.0	7
Gerstein et al. (27)	Germany	Retrospective	28	≥65.0	52.9	6
Lai et al. (28)	USA	Retrospective	1,059	≥65.0	NA	7
Laigle-Donadey et al. (29)	France	Retrospective	19	≥70.0	55.0	5
Kimple et al. (30)	USA	Retrospective	16	≥70.0	37.5	5
Ewelt et al. (31)	Germany	Retrospective	60	≥65.0	50.5	6
Hashem et al. (32)	India	Retrospective	10	≥60.0	70.0	5
Oszvald et al. (33)	Germany	Retrospective	61	≥65.0	52.7	6
Lee et al. (34)	South Korea	Retrospective	11	≥70.0	40.0	5
Pichler et al. (35)	Austria	Retrospective	107	≥60.0	64.7	7
Mukherjee et al. (36)	USA	Retrospective	116	≥70.0	51.5	6
Noorbakhsh et al. (37)	USA	Retrospective	8,152	≥60.0	58.7	7
Hoffermann et al. (38)	Austria	Retrospective	97	≥65.0	58.9	7
Tsang et al. (39)	Canada	Retrospective	181	≥65.0	48.5	7
Zhang et al. (40)	China	Retrospective	70	≥60.0	61.4	6
Flanigan et al. (41)	USA	Retrospective	161	≥65.0	57.8	7
Chen et al. (42)	USA	Retrospective	469	≥66.0	59.4	7

### Overall Survival

**Figure 2:**
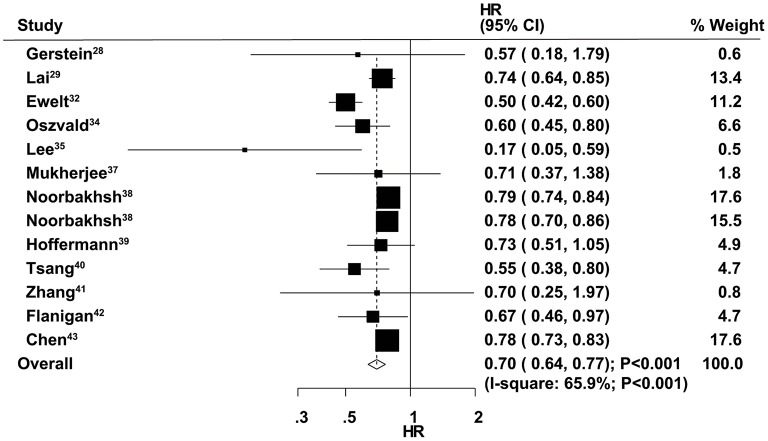
Therapeutic effects of gross total resection (GTR) and subtotal resection (STR) on overall survival (OS) in elderly patients with high-grade glioma.

The therapeutic effects of GTR and STR on OS were obtained from 13 cohorts in 12 studies. Overall, patients who received GTR had a significant improvement in OS compared with the STR group (HR = 0.70, 95% CI = 0.64–0.77, *P* < 0.001; Figure 2). Significant heterogeneity was found among the included studies (*I*^2^ = 65.9%, *P* < 0.001). Sensitivity analysis indicated that the pooled results were stable and not altered by the exclusion of any particular study (Supplementary Material 1). Subgroup analysis suggested a significant improvement in OS in most subsets, except studies conducted in Asia and those with age cutoff ≥70 years and male percentage ≥60.0% (Table 2). Although no significant publication bias for OS by Begg's test was observed (*P* = 0.127), Egger's test indicated a potential publication bias for OS (*P* = 0.024). The summary results were not altered after adjustment for publication bias by the trim-and-fill method (Supplementary Material 2) (43).

**Table 2:** Subgroup analysis of the investigated outcomes.

**Outcomes**	**Subgroups**	**HR or OR and 95%CI**	***P*-value**	**Heterogeneity (%)**	***P*-value for heterogeneity**	***P*-value between subgroups**
Overall survival	**Publication year**					
	Before 2014	0.57 (0.44–0.76)	<0.001	74.8	0.003	<0.001
	2014 or after	0.78 (0.75–0.81)	<0.001	0.0	0.731	
	**Country**					
	Europe	0.57 (0.48–0.68)	<0.001	20.9	0.285	<0.001
	America	0.78 (0.75–0.81)	<0.001	0.0	0.579	
	Asia	0.36 (0.09–1.44)	0.149	65.7	0.088	
	**Sample size**					
	≥100	0.78 (0.75–0.81)	<0.001	0.0	0.579	<0.001
	<100	0.56 (0.46–0.70)	<0.001	32.3	0.194	
	**Age criteria (years)**					
	60–69	0.69 (0.62–0.76)	<0.001	69.3	0.001	0.689
	≥70	0.60 (0.33–1.08)	0.086	65.0	0.057	
	**Percentage male**					
	≥60	0.70 (0.25–1.97)	0.500	–	–	0.943
	<60	0.69 (0.62–0.76)	<0.001	71.5	<0.001	
	**Study quality**					
	High	0.78 (0.75–0.81)	<0.001	0.0	0.573	<0.001
	Low	0.53 (0.45–0.63)	<0.001	5.7	0.380	
3-month mortality	**Publication year**					
	Before 2014	0.67 (0.25–1.81)	0.430	42.1	0.110	0.113
	2014 or after	0.30 (0.15–0.63)	0.001	0.0	0.499	
	**Country**					
	Europe	0.66 (0.28–1.53)	0.333	37.6	0.156	0.209
	America	0.28 (0.07–1.13)	0.074	36.0	0.210	
	Asia	0.21 (0.03–1.38)	0.103	–	–	
	**Sample size**					
	≥100	0.45 (0.10–1.95)	0.282	72.3	0.027	1.000
	<100	0.47 (0.21–1.03)	0.058	16.4	0.305	
	**Age criteria (years)**					
	60–69	0.42 (0.22–0.79)	0.007	22.8	0.255	0.389
	≥70	1.09 (0.07–15.91)	0.950	65.2	0.057	
	**Percentage male**					
	≥60	0.51 (0.15–1.71)	0.274	44.6	0.144	0.441
	<60	0.44 (0.18–1.07)	0.069	39.4	0.143	
	**Study quality**					
	High	0.46 (0.17–1.20)	0.113	58.2	0.066	1.000
	Low	0.49 (0.16–1.51)	0.215	30.4	0.207	
6-month mortality	**Publication year**					
	Before 2014	0.55 (0.32–0.92)	0.022	0.0	0.936	0.035
	2014 or after	0.23 (0.13–0.42)	<0.001	0.0	0.875	
	**Country**					
	Europe	0.48 (0.30–0.78)	0.003	0.0	0.720	0.197
	America	0.26 (0.12–0.55)	<0.001	0.0	0.723	
	Asia	0.16 (0.04–0.72)	0.017	0.0	0.760	
	**Sample size**					
	≥100	0.38 (0.19–0.73)	0.004	30.8	0.236	1.000
	<100	0.38 (0.22–0.67)	0.001	0.0	0.779	
	**Age criteria (years)**					
	60–69	0.37 (0.24–0.55)	<0.001	0.0	0.627	0.616
	≥70	0.51 (0.15–1.78)	0.293	0.0	0.541	
	**Percentage male**					
	≥60	0.48 (0.25–0.90)	0.023	0.0	0.494	0.355
	<60	0.33 (0.20–0.54)	<0.001	0.0	0.730	
	**Study quality**					
	High	0.35 (0.21–0.57)	<0.001	5.7	0.364	0.532
	Low	0.46 (0.23–0.92)	0.027	0.0	0.768	
9-month mortality	**Publication year**					
	Before 2014	0.39 (0.25–0.61)	<0.001	0.0	0.623	0.417
	2014 or after	0.30 (0.17–0.50)	<0.001	0.0	0.814	
	**Country**					
	Europe	0.38 (0.25–0.57)	<0.001	1.8	0.411	0.812
	America	0.32 (0.17–0.61)	0.001	0.0	0.908	
	Asia	0.25 (0.07–0.95)	0.041	0.0	0.476	
	**Sample size**					
	≥100	0.36 (0.20–0.66)	0.001	38.0	0.199	0.681
	<100	0.32 (0.20–0.54)	<0.001	0.0	0.850	
	**Age criteria (years)**					
	60–69	0.37 (0.26–0.53)	<0.001	0.0	0.647	0.295
	≥70	0.19 (0.06–0.62)	0.006	0.0	0.774	
	**Percentage male**					
	≥60	0.42 (0.25–0.71)	0.001	0.0	0.419	0.403
	<60	0.31 (0.20–0.48)	<0.001	0.0	0.824	
	**Study quality**					
	High	0.33 (0.21–0.54)	<0.001	26.1	0.255	0.771
	Low	0.38 (0.21–0.70)	0.002	0.0	0.852	
1-year mortality	**Publication year**					
	Before 2014	0.41 (0.27–0.62)	<0.001	0.0	0.713	1.000
	2014 or after	0.39 (0.23–0.68)	0.001	0.0	0.433	
	**Country**					
	Europe	0.43 (0.29–0.64)	<0.001	0.0	0.713	0.346
	America	0.40 (0.20–0.79)	0.008	0.0	0.573	
	Asia	0.13 (0.03–0.63)	0.011	0.0	0.620	
	**Sample size**					
	≥100	0.46 (0.29–0.72)	0.001	0.0	0.418	0.404
	<100	0.34 (0.21–0.56)	<0.001	0.0	0.790	
	**Age criteria (years)**					
	60–69	0.42 (0.30–0.60)	<0.001	0.0	0.708	0.364
	≥ 70	0.24 (0.08–0.75)	0.014	0.0	0.649	
	**Percentage male (%)**					
	≥60	0.44 (0.26–0.74)	0.002	0.6	0.403	0.627
	<60	0.37 (0.24–0.58)	<0.001	0.0	0.817	
	**Study quality**					
	High	0.44 (0.30–0.66)	<0.001	0.0	0.612	0.364
	Low	0.32 (0.17–0.58)	<0.001	0.0	0.726	

### Three-Month Mortality

**Figure 3:**
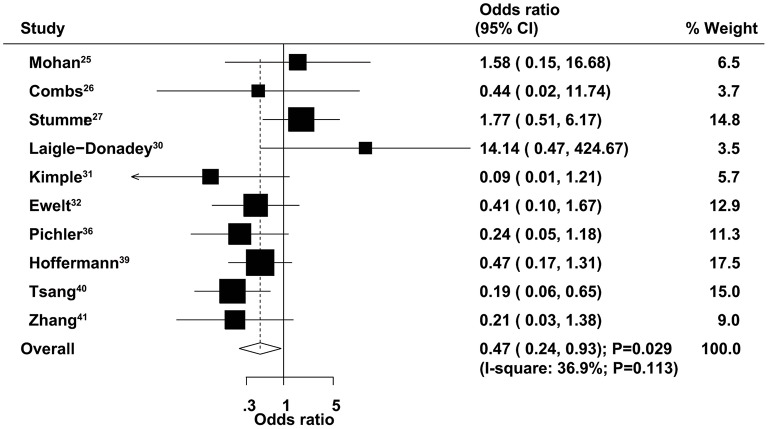
Therapeutic effects of gross total resection (GTR) and subtotal resection (STR) on 3-month mortality in elderly patients with high-grade glioma.

The therapeutic effects of GTR and STR on 3-month mortality were obtained from 10 studies. The summary OR indicated that GTR was associated with a reduced risk of 3-month mortality compared with STR (OR = 0.47, 95% CI = 0.24–0.93, *P* = 0.029; Figure 3), with non-significant heterogeneity across the included studies (*I*^2^ = 36.9%, *P* = 0.113). The summary results were variable in sensitivity analysis due to a marginal 95% CI (Supplementary Material 1). Subgroup analysis indicated that significant differences between GTR and STR in 3-month mortality mainly focused on those studies published in 2014 or after and those that used 60–69 years as the cutoff age (Table 2). There was no significant publication bias for 3-month mortality (*P*-value for Egger's test, 0.688; *P*-value for Begg's test, 0.858) (Supplementary Material 2).

### Six-Month Mortality

**Figure 4:**
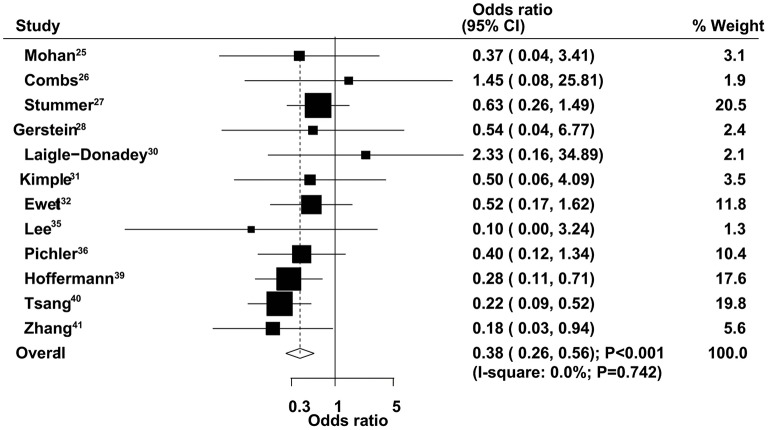
Therapeutic effects of gross total resection (GTR) and subtotal resection (STR) on 6-month mortality in elderly patients with high-grade glioma.

The therapeutic effects of GTR and STR on 6-month mortality were obtained from 12 studies. We found that GTR was associated with a reduced risk of 6-month mortality compared with STR (OR = 0.38, 95% CI = 0.26–0.56, *P* < 0.001), with no evidence of heterogeneity (Figure 4). Sensitivity analysis indicated that the conclusion was not changed after sequential exclusion of individual studies (Supplementary Material 1). Subgroup analysis indicated significant differences between GTR and STR on 6-month mortality in most subsets, except studies that used 70 years of age as the cutoff (Table 2). No significant publication bias for 6-month mortality was detected (*P* value for Egger's test, 0.468; *P* value for Begg's test, 0.537) (Supplementary Material 2).

### Nine-Month Mortality

**Figure 5:**
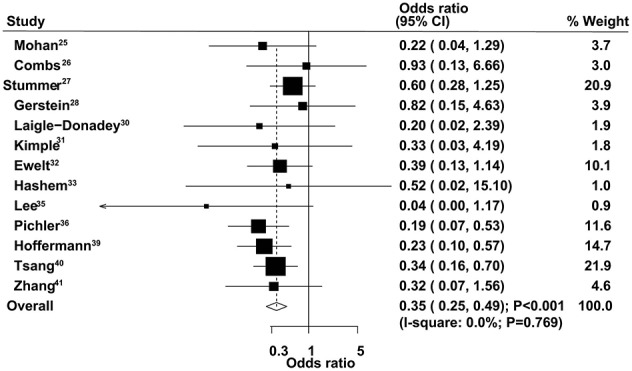
Therapeutic effects of gross total resection (GTR) and subtotal resection (STR) on 9-month mortality in elderly patients with high-grade glioma.

The therapeutic effects of GTR and STR on 9-month mortality were obtained from 13 studies. The summary OS indicated that GTR was associated with a reduced risk of 9-month mortality compared with STR (OR = 0.35, 95% CI = 0.25–0.49, *P* < 0.001), with no evidence of heterogeneity (Figure 5). The pooled results were not altered after sequential exclusion of single studies (Supplementary Material 1). The results of stratified analyses were consistent with the overall analysis in all subsets (Table 2). There was no significant publication bias for 9-month mortality (*P*-value for Egger's test, 0.606; *P*-value for Begg's test, 0.760) (Supplementary Material 2).

### One-Year Mortality

**Figure 6:**
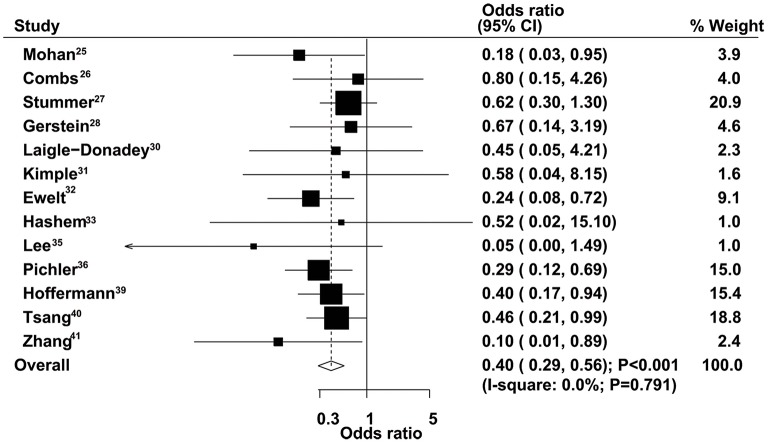
Therapeutic effects of gross total resection (GTR) and subtotal resection (STR) on 1-year mortality in elderly patients with high-grade glioma.

The therapeutic effects of GTR and STR on 1-year mortality were obtained from 13 studies. Patients administered GTR showed a significantly reduced risk of 1-year mortality compared with the STR group (OR = 0.40, 95% CI = 0.29–0.56, *P* < 0.001), with no evidence of heterogeneity (Figure 6). Sensitivity analysis indicated that the pooled results were not changed after excluding any specific single study (Supplementary Material 1). The results of stratified analyses in all subsets were consistent with the overall analysis (Table 2). No significant publication bias for 1-year mortality was detected (*P*-value for Egger's test, 0.277; *P*-value for Begg's test, 0.200) (Supplementary Material 2).

## Discussion

The therapeutic effects of GTR in patients with high-grade glioma have been reported. However, it remains unclear whether GTR is superior to STR for the treatment of elderly patients. This study was based on published reports and explored any potential survival benefits for elderly patients administered GTR or STR. Our comprehensive meta-analysis included 10,815 elderly patients with high-grade glioma in 19 retrospective studies with a wide range of characteristics. The summary results indicated that elderly patients who received GTR had significant improvements in OS and 3-, 6-, 9-month, and 1-year mortality. The therapeutic effects of GTR vs. STR on OS in elderly patients differed by publication year, country, sample size, and study quality, while the effect on 6-month mortality might be affected by publication year.

A previous meta-analysis has compared various extents of tumor resection on the overall and progression-free survival rates in adult GBM patients (13). They pointed out that GTR vs. STR shows significantly reduced mortality rates at 1- and 2-years. Further, the risk of 1-year mortality in patients administered STR was significantly reduced compared with the biopsy group. Moreover, patients treated with GTR or STR showed a significantly reduced risk of mortality at 1- or 2-years compared with the biopsy group. Finally, the latter study found a significant improvement in 1-year disease progression, with no significant effect on 6-month disease progression in the GTR group. However, the above study included both prospective and retrospective studies. In this study, younger GBM patients were included, which could result in greater survival benefits for patients administered GTR. Li et al. performed a meta-analysis based on three randomized controlled trials and three retrospective studies and indicated that GTR significantly improves 1-year OS and 1-year PFS compared with incomplete resection in GBM patients (44). However, numerous studies meeting the inclusion criteria were not included in their analysis. Almenawer et al. compared GTR, partial resection, and biopsy in elderly patients with high-grade glioma and found a greater improvement in survival time, functional recovery, and tumor recurrence rate in patients receiving increasing extents of safe resection (9). However, the latter study used 0.05 as the inspection level, and comparing three types of surgery might increase type I error. Therefore, the current meta-analysis was performed to address the above limitations and evaluate the therapeutic effects of GTR and STR in elderly patients with high-grade glioma.

The summary results indicated a significant improvement in the survival outcomes in elderly patients who received GTR. Although most included studies reported a significant improvement in OS, four reports showed no significant therapeutic advantage of GTR vs. STR (27, 36, 38, 40). This might be due to the small numbers of included patients, and wide 95%CIs were obtained. The significant improvement in the survival outcomes for GTR vs. STR in elderly patients with high-grade glioma could be due to surgical resection-associated tumor load reduction, which creates a favorable environment for postoperative adjuvant therapy. Therefore, patients should be recommended to undergo maximal therapy, including maximal resection, radiotherapy, and chemotherapy, to obtain improved survival.

Subgroup analysis indicated that publication year, country, sample size, and study quality might affect the therapeutic effects of GTR and STR on the survival outcomes. The potential reasons might include: (1) publication year and country are correlated with improved background therapies, including radiotherapy and chemotherapy, and (2) the sample size and study quality could affect the stability of effect estimates and evidence level. Moreover, the age cutoff value of the included patients may have influenced the choice for aggressive therapies. Finally, sex differences for the therapeutic effects of GTR and STR might be due to other lifestyle factors and the prevalence of gene mutations.

The limitations of this meta-analysis should be mentioned: (1) all included studies had a retrospective observational design, which might overestimate the therapeutic effects of GTR in elderly patients; (2) stratified analyses in several subsets only included small numbers of studies, which yielded variable results; (3) the study was based on published articles and unpublished data were not available, which might cause potential publication bias; (4) the detailed characteristics of tumor grade, molecular status, and tumor location were not available in most studies, restricting further stratified analyses based on these factors; and (5) the adjusted factors differed across the included studies, and such factors might play an important role in the prognosis of high-grade glioma.

In summary, this meta-analysis suggested that GTR could significantly improve OS and 3-, 6-, 9-month, and 1-year mortality. Several factors in studies or patients might affect these therapeutic effects, including publication year, country, sample size, and study quality. Comprehensively assessing the therapeutic effects of GTR requires prospective, large sample size, multicenter, and high-quality randomized controlled trials to determine its usefulness in elderly patients with high-grade glioma.

## Data Availability Statement

The raw data supporting the conclusions of this article will be made available by the authors, without undue reservation, to any qualified researcher.

## Author Contributions

QH and HL carried out the studies, participated in collecting data, and drafted the manuscript. PC and HY performed the statistical analysis and participated in its design. PZ helped to draft the manuscript. All authors read and approved the final manuscript.

### Conflict of Interest

The authors declare that the research was conducted in the absence of any commercial or financial relationships that could be construed as a potential conflict of interest.
