# Supplementary material for: Gross Total vs. Subtotal Resection on Survival Outcomes in Elderly Patients With High-Grade Glioma: A Systematic Review and Meta-Analysis
Source: Front Oncol. 2020 Mar 18;10:151. doi: 10.3389/fonc.2020.00151 (PMC7093492; doi:10.3389/fonc.2020.00151)
Supplement: Supplementary file 1 [file Data_Sheet_1.PDF]

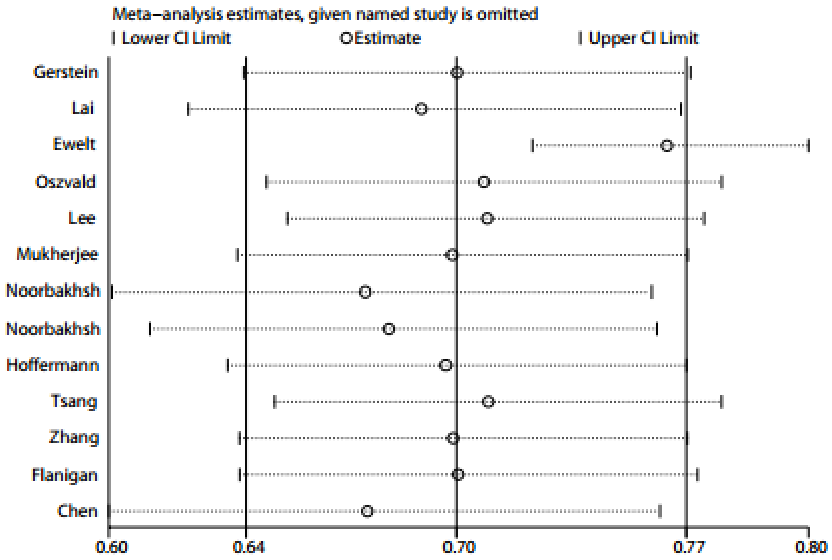

Figure S1. Sensitivity analysis for OS

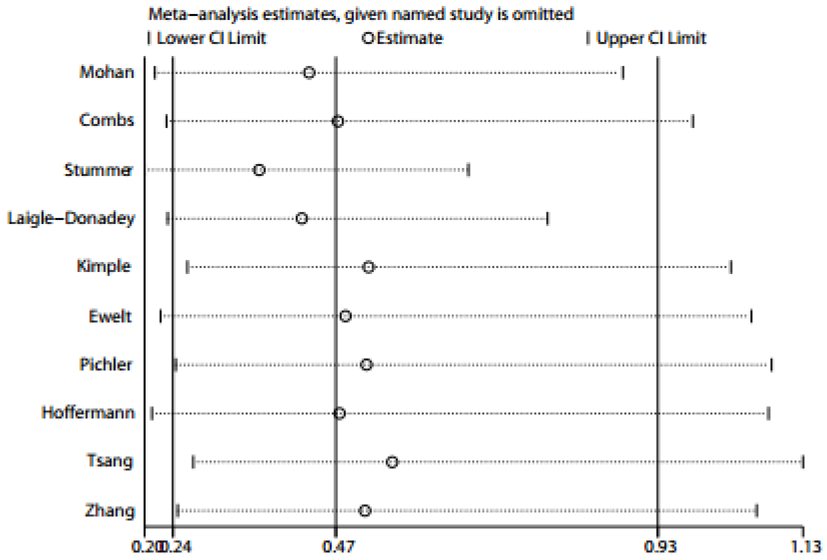

Figure S2. Sensitivity analysis for 3-month mortality

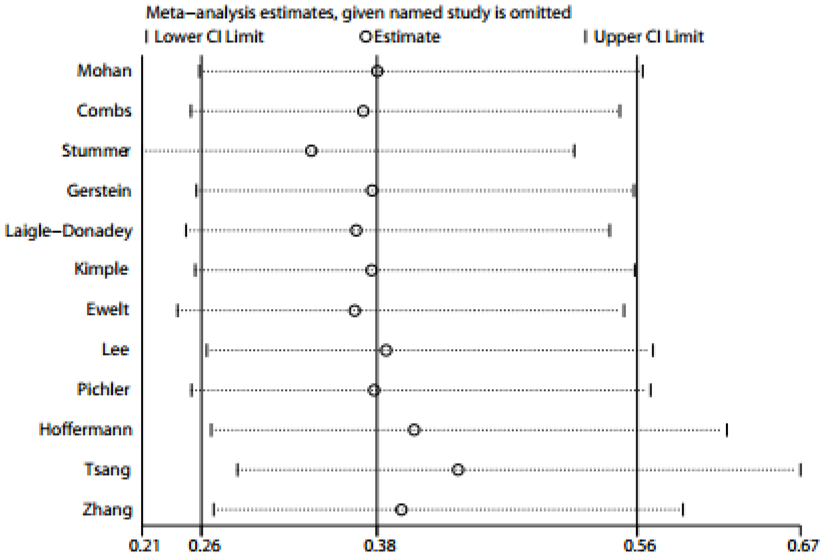

Figure S3. Sensitivity analysis for 6-month mortality

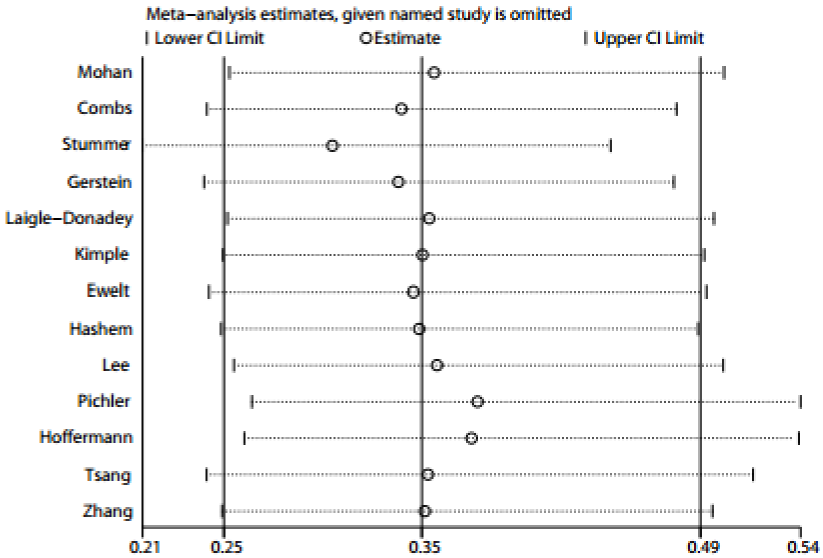

Figure S4. Sensitivity analysis for 9-month mortality

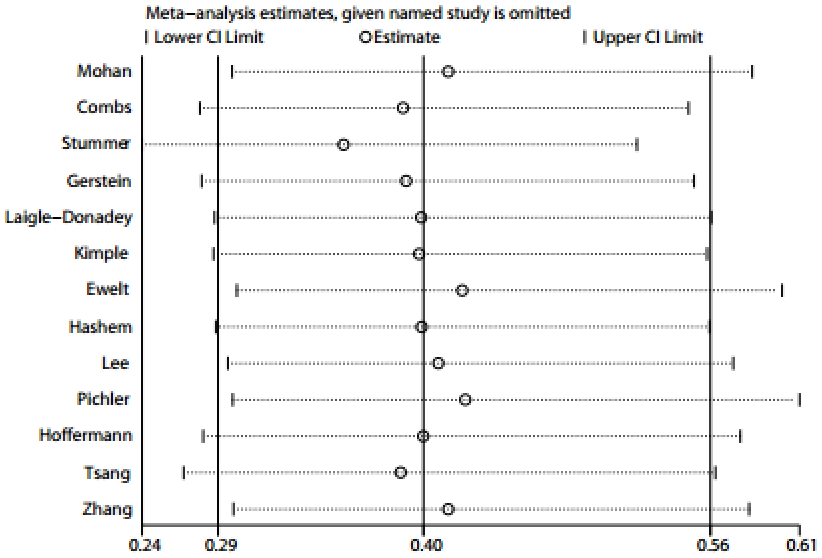

Figure S5. Sensitivity analysis for 1-year mortality
