# Supplementary material for: Gross Total vs. Subtotal Resection on Survival Outcomes in Elderly Patients With High-Grade Glioma: A Systematic Review and Meta-Analysis
Source: Front Oncol. 2020 Mar 18;10:151. doi: 10.3389/fonc.2020.00151 (PMC7093492; doi:10.3389/fonc.2020.00151)
Supplement: Supplementary file 2 [file Data_Sheet_2.PDF]

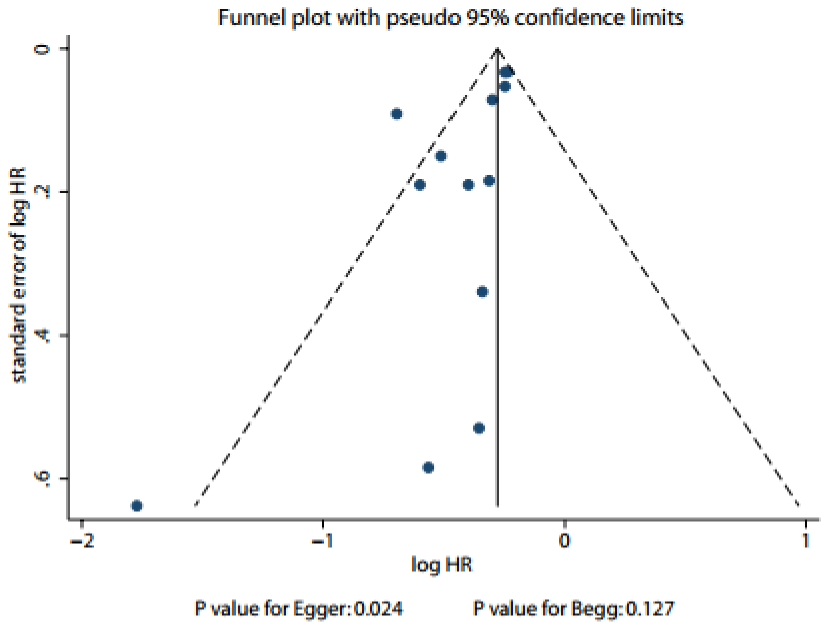

Figure S1. Funnel plot for OS

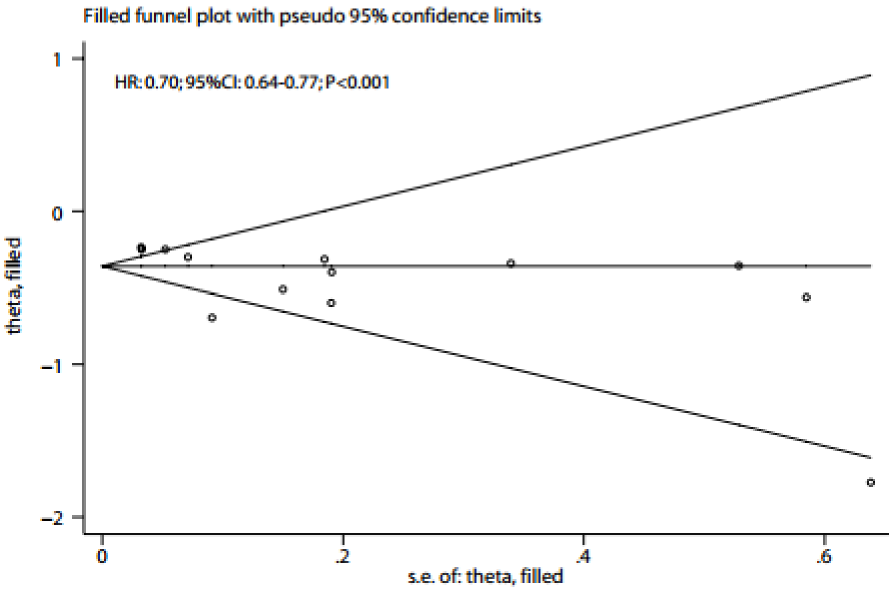

Figure S2. OS adjusted by the trim and fill method

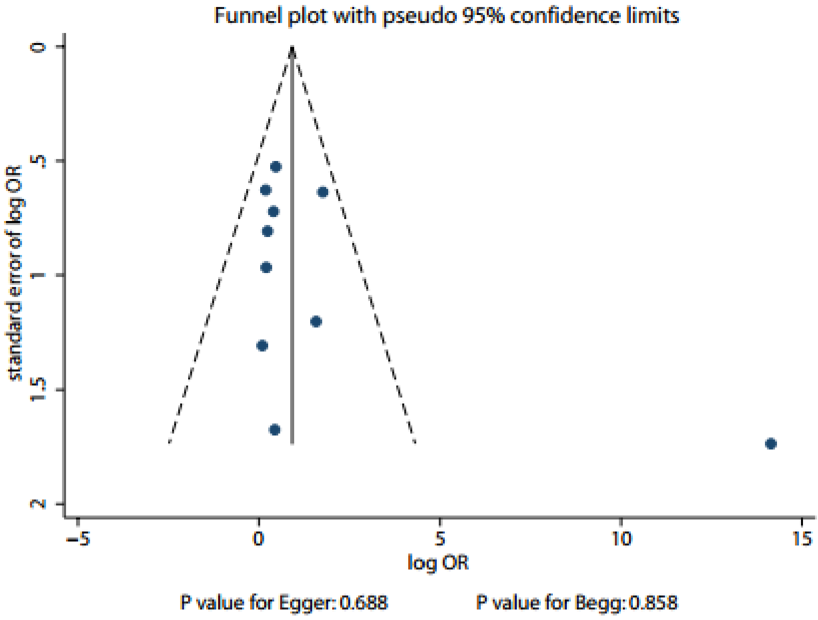

Figure S3. Funnel plot for 3-month mortality

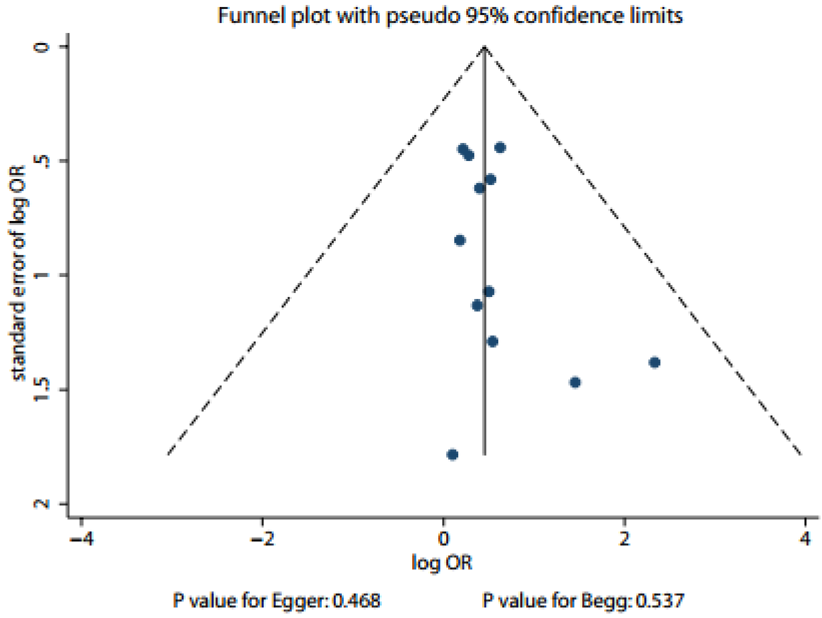

Figure S4. Funnel plot for 6-month mortality

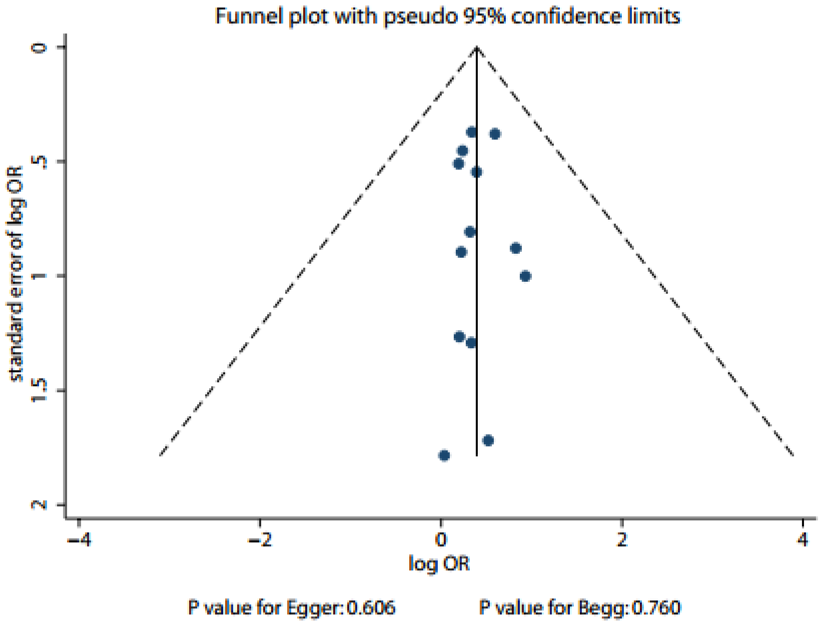

Figure S5. Funnel plot for 9-month mortality

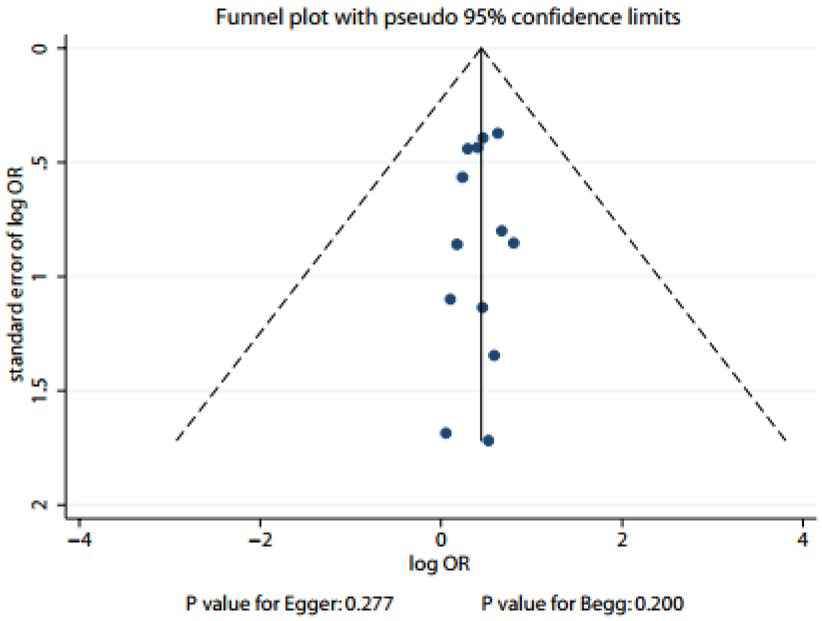

Figure S6. Funnel plot for 1-year mortality
